# Acidic and enzymatic saccharification of waste agricultural biomass for biotechnological production of xylitol

**DOI:** 10.1186/s13065-017-0331-z

**Published:** 2017-10-02

**Authors:** Abdul Ghaffar, Muhammad Yameen, Nosheen Aslam, Fatima Jalal, Razia Noreen, Bushra Munir, Zahed Mahmood, Sadaf Saleem, Naila Rafiq, Sadia Falak, Imtiaz Mahmood Tahir, Muhammad Noman, Muhammad Umar Farooq, Samina Qasim, Farooq Latif

**Affiliations:** 1 Department of Biochemistry, Government College University, Faisalabad, 38000 Pakistan; 2 Department of Zoology and Fisheries, Government College University, Faisalabad, 38000 Pakistan; 3 Department of Chemistry, Government College for Women University, Faisalabad, 38000 Pakistan; 4 School of Pharmacy, University of Faisalabad, Faisalabad, 38000 Pakistan; 5 College of Allied Health Professionals, Government College University, Faisalabad, 38000 Pakistan; 6 Industrial Biotechnology Division, National Institute for Biotechnology and Genetic Engineering (NIBGE), 577, Faisalabad, Pakistan

**Keywords:** Acid hydrolysis, Yeast fermentation, Xylitol yield and productivity

## Abstract

**Background:**

The plant biomass and agro-industrial wastes show great potential for their use as attractive low cost substrates in biotechnological processes. Wheat straw and corn cob as hemicellulosic substrates were acid hydrolyzed and enzymatically saccharified for high xylose production. The hydrolysate was concentrated and fermented by using *Saccharomyces cerevisiae* and *Kluyveromyces* for production of xylitol.

**Results:**

Acid hydrolysis of wheat straw and corn cob in combination with enzymatic hydrolysis showed great potential for production of free sugars from these substrates. *Kluyveromyces* produced maximum xylitol from acid treated wheat straw residues with enzymatic saccharification. The percentage xylitol yield was 89.807 g/L and volumetric productivity of 0.019 g/L/h. *Kluyveromyces* also produced maximum xylitol from corn cob acid hydrolyzed liquor with xylitol yield 87.716 g/L and volumetric productivity 0.018 g/L/h.

**Conclusion:**

Plant and agro-industrial biomass can be used as a carbohydrate source for the production of xylitol and ethanol after microbial fermentation. This study revealed that wheat straw acid and enzyme hydrolyzed residue proved to be best raw material for production of xylitol with *S. cerevisiae*. The xylitol produced can be utilized in pharmaceuticals after purification on industrial scale as pharmaceutical purposes.

## Background

Lignocellulosic agricultural biomass accounts for more than 60% of the total biomass produced in the form of wheat straw, rice straw, corn cob, corn fibers, para grass, kallar grass and maize stover. Tons of agro-industrial residues are generated annually in agricultural country like Pakistan. This residue contains significant amount of biochemical fractions like cellulose, hemicellulose and lignin to be converted into many valuable products for food and pharmaceuticals [1–3]. Cellulose, hemicellulose and lignin can be acid and enzymatically hydrolyzed and fermented into glucose, mannose, xylose, xylitol, arabinose, acetic acid, glycerol, methanol, methane, butanol, furfural, hydroxyl methyl furfural, 5-hydroxyl methyl furfural, succinic acid and many other products [4–6].

The acid and enzymatic hydrolysis break covalent bonds, hydrogen bonds, van der Waals forces and various intermolecular bridges between sugars. Agricultural biomass such as corn cob and wheat straw was acid hydrolyzed with mild acid 72% H_2_SO_4_ for production of xylitol [7]. The acid/enzyme (H_2_SO_4_, cellulase and xylanase) treatments release sugars which are converted into xylitol after microbial fermentation [3]. Xylitol is identified as one of the twelve high value added chemicals which can be produced from lignocellulosic agricultural biomass through cost effective methods [8].

Xylitol is a five carbon polyalcohol sugar having vast applications in food and pharmaceutical industry. It plays an important role to economic grooming of an agricultural country [9, 10]. It is a sweetening powder like glucose, xylose, fructose and sucrose. Xylitol can be transported into the cell without insulin and can be used as sugar substitute with low calories. It inhibits the growth of tooth decaying microorganisms [11, 12]. It fights against bacterial growth, particularly to *Streptococcus*. It is digested slowly in the large intestine, reduces the bacterial growth in stomach and plays an important role in oral health [13, 14]. It can be used for children to prevent middle ear infection (otitis media) and upper respiratory disorders [9, 11, 13, 15]. Xylitol helps in the treatment of hypoglycemia, as a sweetener for diabetic patients [11, 12]. It has no side effect in the human body [16].

The chemical production of xylitol is much expensive due to the requirement of high values of temperature and pressure, therefore, microbial production through fermentation process is more attractive. This process is environmental friendly and doesn’t need noxious catalyst. The yeast strain *Candida boidinii, Candida parapsilasis, Saccharomyces cerevisiae, Pichia stipites*, *Kluyveromyces marxianus* and *Debaryomyces hansenii* have been used for xylitol production from different waste agro-biomasses [4, 17–19]. *Candida tropicalis* has been used for the production of xylitol from corn cob and sugarcane bagasse [20]. Acid treated corn cob and rice straw have been previously fermented into xylitol by using *C. magnolia, C. guilliermondii and S. cerevisiae* [21–25]. This study reports xylitol production by *S. cerevisiae* and *Kluyveromyces* from easily available biomasses like wheat straw and corn cob.

## Methods

### Substrate collection and acid hydrolysis

Waste agricultural biomass in the form of wheat straw and corn cob was obtained after its seasonal cultivation from local agricultural fields of Faisalabad (Pakistan) and Rafhan Maize Products (Pvt) Limited, Faisalabad (Pakistan). It was dried in oven at 55 °C for 24 h and ground to a particle size of 2 mm. The acid hydrolysis of complex polysaccharides present in wheat straw and corn cob was carried out using 72% H_2_SO_4_ for breakdown of lignocellulosic biomass into different sugar fractions. Wheat straw and corn cob (200 g each) were acid treated with 1% (v/v) of 72% H2SO4 at a ratio of 1 g substrate to 5 mL acidic solution in 2 litter Erlenmeyer flask separately and autoclaved at 121 °C for 1 h [15].

### Enzymatic saccharification of raw material

The acid hydrolysis contents were filtered through cheese cloth to separate hydrolysate and residue. The hydrolysate was diluted to 1 L with distilled water and the residue was dried at room temperature. White precipitates formed during neutralization of hydrolysate with Ca(OH)_2_ were removed through filtration. One hundred milliliter of hydrolysate was treated with activated charcoal to remove other impurities. The treated hydrolysate was heated at 80 °C for 30 min. The mixture was cooled at room temperature and filtered using starch powder bed. The total dissolved solids (TDS) were calculated in the liquor.

Acid treated hemicellulosic hydrolysate and residues were enzymatically saccharified using cellulase and xylanase (10–20 U each). The enzymes were added separately to 10 mL hydrolysate in 250 mL Erlenmeyer flask along with 25 mL of 0.1 M citrate buffer and incubated in reciprocal shaker at 50 °C at 120 rpm for 24 h. The sugar contents in hydrolysate and residue of corn cob and wheat straw were determined using HPLC [26].

### Xylitol production

Acid and enzyme treated wheat straw and corn cob hydrolysate and residue were used separately for xylitol production by the fermentation of *S. cerevisiae* (hexose fermenting yeast) and *Kluyveromyces* (pentose fermenting yeast). *Kluyveromyces* and *S. cerevisiae* were obtained from Industrial Biotechnology Laboratory, National Institute for Biotechnology and Genetic Engineering (NIBGE), Faisalabad, Pakistan and growing cultures were stabilized through various cycles for uniform growth. Universal yeast media (yeast extract 10 g/L, peptone 20 g/L, dextrose 20 g/L, agar 15 g/L for 1 L) was used to harbor the yeast strains at 37 °C in an incubator. The inoculum for each yeast was prepared in 500 mL Erlenmeyer flask using 200 mL distilled water, 0.5% (NH_4_)_2_SO_4_ and MgSO_4_·7H_2_O, 0.05% KH_2_PO_4_, 0.01% CaCl_2_·2H_2_O, 0.1% yeast extract and 3% d-xylose in an incubator shaker at 37 °C and 120 rpm for 12 h [OD_600_ = 1.3–1.5 (10^6^ spores/mL)]. Sterilized substrates, treated with acid and enzyme were inoculated with 10^5^–10^6^ cells/mL separately. All these flasks were placed in shaking incubator at 30 °C (120 rpm). Samples were obtained after 0, 4, 8, 12, 24, 48 and 72 h of fermentation for further analysis [15].

### Analysis of fermented products

The acid/enzyme hydrolysis hemicellulosic hydrolysate and residue after microbial fermentations were analyzed to determine the concentration of sugars, xylitol and ethanol using HPLC.

### HPLC system and conditions

High performance liquid chromatography system of Perkin Elmer (USA) equipped with BioRad Aminex HPX-87H column with corresponding guard column and variable wavelength diode array detector was used to determine the concentration of sugars, xylitol and ethanol. The mobile phase consisted of 0.001 *N* sulphuric acid. A series of calibration standards containing xylose, glucose and xylitol were prepared and filtered through 0.2 µm membrane filter. Twenty microliters of each standard was analyzed by HPLC at a flow rate of 0.6 mL/min, column temperature 65–75 °C for a retention time of 15 and 20 min. The samples were appropriately diluted, filtered and analyzed in the same way.

### Statistical analysis

The obtained HPLC chromatogram results were statistically analyzed for calculation of % yield, volumetric productivity and Qs.

## Results and discussion

Wheat straw and corn cob on acid and enzyme hydrolysis produced sufficient amount of carbohydrates for use as substrate to produce xylitol. The enzyme coupled acid hydrolysis showed promising increase in liberating carbohydrate monomers from cellulose and hemicellulose. Xylose, glucose and cellobiose were the principal components in acid hydrolysate of wheat straw and corn cob (Table 1).

**Table 1:** Carbohydrate contents of wheat straw and corn cob acid hydrolyzed liquor

Substrate	Xylose g/L	Glucose g/L	Cellobiose g/L
W.S.A.H.L*	25.183	3.594	0.058
C.C.A.H.L**	25.039	2.350	0.014

W.S.A.H.L*, wheat straw acid hydrolyzed liquor; C.C.A.H.L**, corn cob acid hydrolyzed liquor

Wheat straw and corn cob acid hydrolysis of liquor showed that xylose was present as a major sugar fraction 25.183 and 25.039 g/L, respectively. Second major fraction found in both the liquors was glucose 3.594 and 2.350 g/L respectively. Glucose was present in higher concentration in wheat straw than corn cob. The other sugar fractions found in minor amount was cellobiose 0.058 and 0.014 g/L, respectively.

**Fig. 1:**
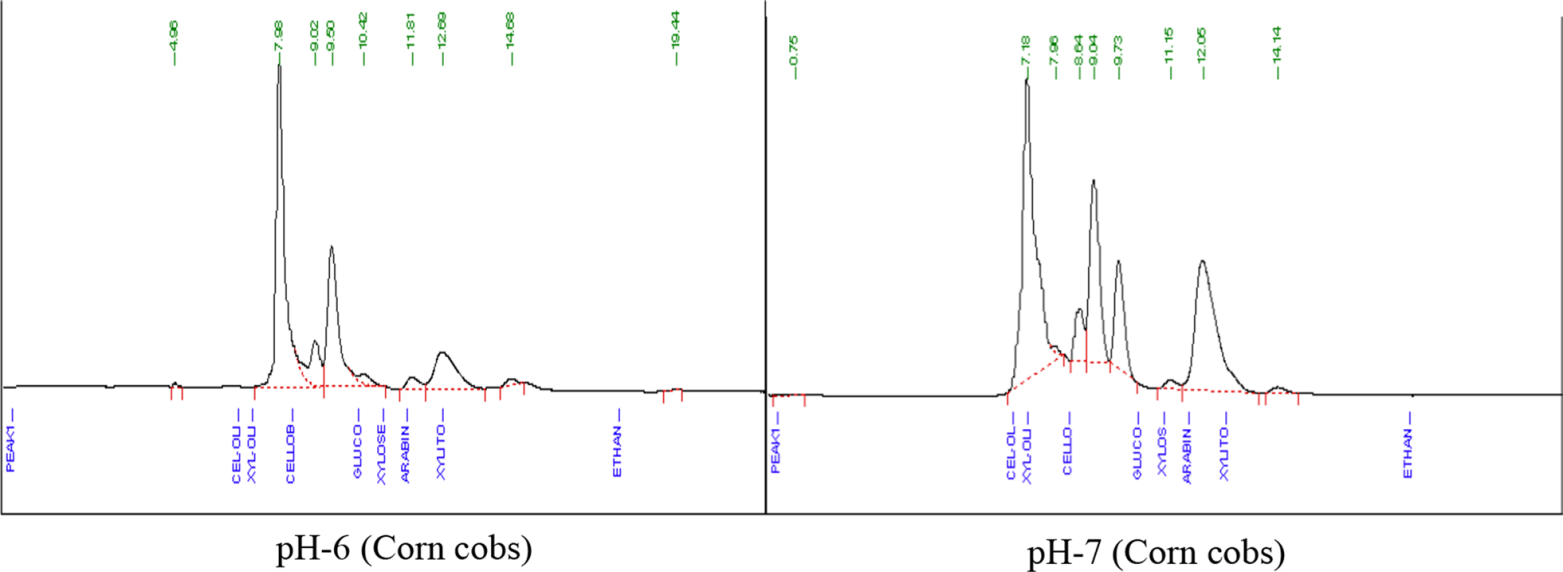
HPLC Chromatogram carbohydrate contents of wheat straw and corn cobs acid hydrolyzed and enzymatic scarified residues fermented to produce xylitol

Acid and enzyme hydrolysis of wheat straw liquor produced xylose 27.772 g/L, glucose 3.947 g/L and cellobiose 0.209 g/L which shows that xylose was the major sugar component (Table 2). The xylose was converted into xylitol by pentose utilizing yeast *Kluyveromyces*. The xylitol and glucose were used by the yeast as carbohydrate source for its metabolism, growth and energy production. *Kluyveromyces* yeast on fermentation produced xylitol 3.659 g/L with yield percentage of 65.56 g/L and volumetric productivity of 0.014 g/L/h after 48 h (Fig. 1). The rate of substrate consumption (Qs) showed that the yeast used 0.116 g/L of xylose during the microbial metabolism to ferment the xylose to xylitol.

**Table 2:** Carbohydrate contents of wheat straw and corn cob acidic and enzymatic hydrolyzed liquor and residue fermented to produce xylitol with microorganisms (a-*Kluyveromyces* and b-*S. cerevisiae*) at pH 6 and 7, respectively

Substrates	Treatments	Free sugars			Xylitol					pH
	Acid and enzyme	Xylose (I) g/L	Glucose g/L	Cellobiose g/L	Yeast	Qs g/L/h	Xylitol (P) g/L	Xylitol yield %	Productivity g/L/h	
Wheat straw	WS.A,E.H. L	27.772	3.947	0.209	A	0.116	3.659	65.561	0.014	7
					B	0.076	3.064	83.578	0.017	7
	WS.A,E.H. R	27.382	3.044	0.005	A	0.480	19.639	85.242	0.018	7
					A	0.570	24.592	89.807	0.019	6
Corn Cob	CC.A,E.H. L	28.894	0.017	0.237	A	0.130	5.513	87.716	0.018	7
					B	0.227	4.508	41.282	0.009	7
	CC.A,E.H. R	19.0395	3.744	0.045	A	0.403	3.5924	18.542	0.004	7
					A	0.374	5.687	31.596	0.007	6

I, initial xylose concentration; P, product; Qs, rate of substrate consumption; A, *Kluyveromyces*; B, *S. cerevisiae*

*Saccharomyces cerevisiae* on fermentation in same conditions produced xylitol 3.064 g/L having yield percentage of 83.578 g/L and volumetric productivity of 0.017 g/L/h after 48 h. The Qs showed that the yeast used 0.076 g/L/h of xylose during the microbial metabolism to ferment xylose to xylitol.

Wheat straw acid and enzyme hydrolyzed residue contained xylose 27.382 g/L and glucose 3.044 g/L which showed xylose as a major sugar fraction. This xylose was converted to xylitol 19.639 g/L with yield percentage of 85.242 g/L and volumetric productivity of 0.018 g/L/h after 48 h by *Kluyveromyces* at pH 7. The Qs showed that 0.480 g/L/h of xylose was used by yeast during the metabolism to ferment xylose to xylitol (Table 2).

*Kluyveromyces* produced xylitol 24.592 g/L from acid and enzyme hydrolyzed wheat straw residue with yield percentage of 89.807 g/L and volumetric productivity of 0.019 g/L/h after 48 h of fermentation at pH 6. The Qs showed that the microorganism used 0.570 g/L/h of xylose for production of xylitol.

Corn cob acid and enzyme hydrolyzed liquor contained xylose and glucose 28.894 and 0.017 g/L, respectively. *Kluyveromyces* upon fermentation produced xylitol yield percentage of 87.716 g/L and volumetric productivity of 0.018 g/L/h after 48 h. The Qs show that the microorganism used 0.130 g/L of xylose for production of xylitol. *S. cerevisi*ae fermentation of corn cob acid and enzyme hydrolyzed liquor produced xylitol yield percentage of 41.282 g/L and volumetric productivity of 0.009 g/L/h in same time period. The Qs show that 0.227 g/L/h xylose were used by yeast to ferment xylose to xylitol.

Corn cob acidic and enzymatic hydrolysis residue produced xylitol yield percentage of 31.596 g/L and volumetric productivity of 0.007 g/L/h after 48 h of fermentation with yeast *Kluyveromyces* at pH 6. The Qs show that the microorganism used 0.374 g/L/h of xylose during convwersion of xylose to xylitol. However, at pH 7, *Kluyveromyces* produced xylitol yield percentage of 18.542 g/L and volumetric productivity of 0.004 g/L/h with Qs 0.403 g/L/h.

## Discussion

The aim of this study was to evaluate the utilization of agriculture biomass for the production of value added products like xylitol, beneficial for health, environment and economy. Wheat and corn are major crops of Pakistan which produce a large amount of waste residues. Xylose and glucose were the major sugar fractions (25.183 and 3.594 g/L) in wheat straw acid hydrolysate. The enzyme saccharification of this hydrolysate raised the concentrations of xylose up to 27.772 g/L. The concentration of xylose was reported to be 37 g/L when wheat straw was treated with H_2_SO_4_ [27].

Corn cob acid hydrolysate also produced almost similar amounts of xylose (25.039 g/L) with a less amount glucose (2.350 g/L). However, xylanase saccharification of corn cob acid hydrolysate increased the concentration of xylose up to 28.894 g/L. The concentration of xylose was reported 23.3 g/L when substrate treated with acid H_2_SO_4_ which showed relatively low amount of xylose after acid and enzymatic hydrolysis [28]. The xylitol percentage yield from wheat straw acid and enzyme hydrolysate was 65.561 g/L by yeast *Kluyveromyces* as compared to xylitol 0.59 g/g when substrate was fermented by yeast strain *C. guilliermondii* FTI 20037 [22]. Similar results have been reported in another study for production of almost equal amount of xylitol after 48 h of fermentation under specific conditions [27].

Wheat straw acid and enzyme hydrolysate liquor fermented with *S. cerevisiae*, produced xylitol 83.578 g/L and volumetric productivity of 0.017 g/L/h compared to xylitol 13.7 g/L when wheat straw was fermented by *Debaryomyces hansenii* [28]. The present study reveals efficient production of xylitol from wheat straw acid and enzyme hydrolyzed liquor by *S. cerevisiae* [29]. The microorganism being hexose fermenting yeast also produced ethanol 38.576 g/L and volumetric productivity of 0.803 g/L/h (Ghaffar A. unpublished results). The wheat straw acid and enzyme hydrolyzed residue showed maximum percentage xylitol yield of 89.810 g/L and volumetric productivity 0.0187 g/L/h after 48 h fermentation at pH 6 and 37 °C by *Kluyveromyces*. Corn cob acid and enzyme hydrolyzed liquor showed promising results for production of xylitol percent yield of 87.716 g/L and volumetric productivity of 0.0183 g/L/h by *Kluyveromyces* at pH 7. The present study shows high amount of xylitol as compared to 0.71 g/g xylitol using yeast strain *S. cerevisiae* while 0.50 g/g of xylitol from corn cob with *C. tropicalis* after 72 h fermentation [30].

## Conclusion

The present study reported the comparison of two substrates (wheat straw and corn cob) and two yeasts (*Kluyveromyces* and *S. cerevisiae*) for xylitol production. Wheat straw acid and enzyme hydrolyzed residue was better xylitol producing substrate for *S. cerevisiae* followed by corn cob acid and enzyme hydrolyzed liquor for *Kluyveromyces.* The results proved that *S. cerevisiae* (hexose fermenting yeast) give high yield and volumetric productivity of xylitol and ethanol than *Kluyveromyces* (a pentose fermenting yeast), respectively. The effect of pH on *Kluyveromyces* showed that the xylitol yield and productivity was higher under pH 6 than pH 7. This may be due to additional utilization of glucose for production of xylitol using hexose monophosphate shunt. The acid treated residue after enzymatic saccharification is first time reported for yeast fermentation to produce xylitol. The xylitol produced can be utilized further after the purification on industrial scale for the pharmaceutical purposes.

## Abbreviations

C.C.A.H.L: corn cobs acid hydrolyzed liquor
C.C.A.H.R: corn cobs acid hydrolyzed residue
C.C.A.E.H.L: corn cobs acid and enzyme hydrolyzed liquor
C.C.A.E.H.R: corn cobs acid and enzyme hydrolyzed residue
W.S.A.H.L: wheat straw acid hydrolyzed liquor
W.S.A.H.R: wheat straw acid hydrolyzed residue
W.S.A.E.H.L: wheat straw acid and enzyme hydrolyzed liquor
W.S.A.E.H.R: wheat straw acid and enzyme hydrolyzed residue
Yeast (A): *Kluyveromyces* (pentose fermenting yeast)
Yeast (B): *S. cerevisiae* (hexose fermenting yeast)
